# Bifunctional protein ArsR^M^ contributes to arsenite methylation and resistance in *Brevundimonas* sp. M20

**DOI:** 10.1186/s12866-023-02876-z

**Published:** 2023-05-17

**Authors:** Congcong Li, Gongli Zong, Xi Chen, Meixia Tan, Wenhui Gao, Jiafang Fu, Peipei Zhang, Bing Wang, Guangxiang Cao

**Affiliations:** 1 Shandong Quancheng Test & Technology Limited Company, Ji’nan, 250101 China; 2 Biomedical Sciences College & Shandong Medicinal Biotechnology Centre, Shandong First Medical University & Shandong Academy of Medical Sciences, Ji’nan, 250062 China; 3 NHC Key Laboratory of Biotechnology Drugs, Shandong Academy of Medical Sciences, Ji’nan, 250117 Shandong China

**Keywords:** *Brevundimonas* sp., Arsenic resistance, *Ars* cluster, Bifunctional protein, Inorganic arsenic detoxify

## Abstract

**Background:**

Arsenic (As) with various chemical forms, including inorganic arsenic and organic arsenic, is the most prevalent water and environmental toxin. This metalloid occurs worldwide and many of its forms, especially arsenite [As(III)], cause various diseases including cancer. Organification of arsenite is an effective way for organisms to cope with arsenic toxicity. Microbial communities are vital contributors to the global arsenic biocycle and represent a promising way to reduce arsenite toxicity.

**Methods:**

*Brevundimonas* sp. M20 with arsenite and roxarsone resistance was isolated from aquaculture sewage. The *arsHRNBC* cluster and the *metRFHH* operon of M20 were identified by sequencing. The gene encoding ArsR/methyltransferase fusion protein, *arsR*^*M*^, was amplified and expressed in *Escherichia coli* BL21 (DE3), and this strain showed resistance to arsenic in the present of 0.25–6 mM As(III), aresenate, or pentavalent roxarsone. The methylation activity and regulatory action of ArsR^M^ were analyzed using Discovery Studio 2.0, and its functions were confirmed by methyltransferase activity analysis and electrophoretic mobility shift assays.

**Results:**

The minimum inhibitory concentration of the roxarsone resistant strain *Brevundimonas* sp. M20 to arsenite was 4.5 mM. A 3,011-bp arsenite resistance *ars* cluster *arsHRNBC* and a 5649-bp methionine biosynthesis *met* operon were found on the 3.315-Mb chromosome. Functional prediction analyses suggested that ArsR^M^ is a difunctional protein with transcriptional regulation and methyltransferase activities. Expression of ArsR^M^ in *E. coli* increased its arsenite resistance to 1.5 mM. The arsenite methylation activity of ArsR^M^ and its ability to bind to its own gene promoter were confirmed. The As(III)-binding site (ABS) and *S*-adenosylmethionine-binding motif are responsible for the difunctional characteristic of ArsR^M^.

**Conclusions:**

We conclude that ArsR^M^ promotes arsenite methylation and is able to bind to its own promoter region to regulate transcription. This difunctional characteristic directly connects methionine and arsenic metabolism. Our findings contribute important new knowledge about microbial arsenic resistance and detoxification. Future work should further explore how ArsR^M^ regulates the *met* operon and the *ars* cluster.

**Supplementary Information:**

The online version contains supplementary material available at 10.1186/s12866-023-02876-z.

## Introduction

Arsenic, classified as a Group 1 human carcinogen by the International Agency for Research on Cancer, is the most prevalent water and environmental toxin [1], and ranks top of the US Priority List of Hazardous Substances. Arsenic compounds enter the biosphere from geochemical sources and anthropogenic sources. Herbicides, growth promoters for farm animals, the semiconductor industry, and other industrial sources contribute to arsenic contamination [2]. Humans are exposed to arsenic daily [3], mostly from food and water supplies. This exposure lead to numerous diseases, including cardiovascular and peripheral vascular diseases, neurological disorders, diabetes mellitus, chronic kidney disease [4], and cancer [3, 4]. In addition, low birth rate, fetal death, and delayed infant development are closely associated with arsenic exposure during pregnancy [5].

In the environment, inorganic arsenic exists in various chemical forms, including arsenate [As(V)], arsenite [As(III)], elemental arsenic [As(0)], and arsine [As(− 3)]. The predominant forms in oxic and reducing environments are As(V) and As(III), respectively [2]. Organic arsenic compounds include pentavalent roxarsone [Rox(V)], methylarsenate [MAs(V)], trivalent roxarsone [Rox(III)] [6], and methylarsenite [MAs(III)] [7]. In the past, the organoarsenic compound roxarsone was extensively used as a organoarsenic feed additive for poultry. Although its use has been forbidden in many countries in the world, residues of roxarsone in the environment still pollute waterways and lead to the enrichment of arsenic-tolerant bacteria [8, 9]. These relatively benign organic arsenic compounds including roxarsone, however, are largely degraded into more toxic inorganic forms after they are introduced into the environment [2]. As an important part of the ecosystem, microbial communities are an vital contributors to the global arsenic biocycle [10].

Arsenic resistance (*ars*) genes that confer resistance to arsenic and various organic arsenic compounds, have been identified in plasmids and/or chromosomes of various prokaryotes and eukaryotes [11–15]. *ars* genes are usually present in clusters, and at least one arsenic resistance system seems to be a necessary component of the genome in prokaryotic species, because canonical *arsRBC* and its variants appear to be quite common in bacterial and archaeal species [16]. ArsR, is a SmtB/ArsR family of metalloregulatory proteins that controls the transcription of the *ars* operon [16, 17]. Acr3 and ArsB are responsible for pumping As(III) from the cytosol across the cytoplasmic membrane into the periplasm or extracellular medium [18]. ArsC, an As(V) reductase, reduces intracellular As(V) to As(III) [19]. In organisms, As(III) tolerance and detoxification can be achieved by efflux and methylation [3, 13, 14]. ArsM, an *S*-adenosylmethionine (SAM) methyltransferase, catalyzes the methylation of As(III) using SAM as the substrate in microbes [2]. ArsH is an organoarsenical oxidase that confers resistance to trivalent forms of monosodium methanearsonate and roxarsone [20, 21].

In this study, a roxarsone-resistant strain, M20, was isolated from aquaculture sewage and identified as *Brevundimonas* sp. We characterized an *ars* cluster, *arsHRNBC*, with a novel gene arrangement in strain M20. Meanwhile, we identified a novel fusion protein, ArsR^M^, was found to connect arsenite methylation and methionine metabolism. Thus, our results reveal the mechanism of a novel pathway of arsenite resistance and a potential method by which methylation can detoxify arsenite.

## Materials and methods

### Isolation of the roxarsone-resistant strain ***Brevundimonas*** sp. M20.

Aquaculture sewage was sampled from the influx of a wastewater treatment facility in Shandong Province, China. The sewage samples were diluted and spread onto Luria–Bertani (LB)-agar plates (0.5% w/v yeast extract, 1% w/v tryptone, 1% w/v sodium chloride, 2% w/v agar) containing 30 µM roxarsone (filtered through a 0.22 µM micro-filtration membrane) (Sigma Co., Shanghai, China), which were then incubated at 28 °C for 24 h. All colonies with different phenotypes on the plate were selected and cultivated three to five consecutive times on LB-agar medium containing 30 µM roxarsone to obtain pure cultures of single colonies. One colony, named M20 (Table S1), was selected and grown in pure culture for further study.

### Whole-genome sequencing and genomic analysis

The genome of strain M20 was sequenced using the Nanopore and BGISEQ-500 platform (BGI, Wuhan, China) and assembled using Unicycler software [22]. Additional genome annotation was performed using tools at the RASTtk server [23] and the Pathosystems Resource Integration Center (PATRIC) server [24]. Multisequence comparison was carried out using Clustal Omega [25] and ESPript [26]. Phylogenetic affiliation analysis of strain M20 was performed based on its genome sequence, and a whole-genome phylogenetic tree was constructed using the PATRIC server [24].

### Bioinformatic analysis of arsenic resistance genes and proteins

The genetic contexts of the *arsHRNBC* cluster and the *metRFHH* operon were compared using BLASTn. Comparison of ArsR^M^ and ArsM orthologs was conducted using Blastp, and multisequence comparisons were conducted using Clustal Omega and ESPript. Homology models of ArsR^M^ were constructed using Discovery Studio 2.0 [27].

### Expression and purification of fusion protein ArsR^M^

The *arsR*^*M*^ gene was amplified by PCR using *arsR*^*M*^-His-F/R as primers (Table S2) and the genomic DNA of M20 as the template. The purified *arsR*^*M*^ fragment was cloned into pMD18-T and verified by sequencing. *Nde*I and *Xho*I were used to digest the recombinant plasmid. The *arsR*^*M*^ gene fragment was then inserted into digested pET-15b. The resulting plasmid, pET-arsRM, was transformed into *Escherichia coli* BL21 (DE3), to produce *E. coli* strain ARM3 (Table S1). When the *E. coli* ARM3 culture reached an OD_600 nm_ of 0.6, 1.0 mM isopropyl-β-D-1-thiogalactopyranoside (IPTG) was added to induce the expression of the His-tagged ArsR^M^ fusion protein. After incubation at 28°C for 4 h, the His-tagged ArsR^M^ was purified using a Ni-NTA-Sefinose column (Sangon Biotech Co., Shanghai, China) and then analyzed by 10% SDS-PAGE as previously described [28].

### Determination of minimum inhibitory concentrations

The M20 strain was cultured on LB-agar plates to determine the minimum inhibitory concentrations (MICs) of Rox(V), NaH_2_AsO_3_ [As(III)], and NaH_2_AsO_4_ [As(V)]. First, strain M20 was activated on an LB-agar plate and single colonies were individually transferred into LB medium and cultured overnight at 28 °C with shaking at 180 rpm. OD_600 nm_ of the culture was adjusted to 0.1, and then it was diluted 100-fold with LB medium. Diluted culture was streaked on LB-agar plates containing 0.25–6 mM (0.25-mM increment) of filtered As(III), As(V), or Rox(V). The plates were cultured at 28 °C for 3 days. MICs toward *E. coli* ARM3 were tested in the presence and absence of IPTG (0.5mM). *E. coli* BL21 (DE3) harboring vector pET-15b in the presence of IPTG was used as the negative control.

### Molecular simulation and electromobility shift assays (EMSAs)

The interactions between ArsR^M^ and its substrates As(III) and SAM were analyzed using the CDOCKER protocol of Discovery Studio 2.0 [27]. Molecular docking between ArsR^M^ and its target gene was analyzed by using the ZDOCKER protocol of Discovery Studio 2.0 with the *arsR*^*M*^ promoter region as the ligand. The interaction between ArsR^M^ and its gene target was verified by electromobility shift assays (EMSAs). The promoter region of the *metRFHH* operon was divided into 80- to 120-bp fragments, which were used to design three short oligonucleotide probes (Table S2). Biotin-labeled oligonucleotide probes were synthesized by Sangon. The EMSAs were conducted as previously described [28, 29].

### Methyltransferase activity analysis of ArsR^M^

The ability of ArsR^M^ to methylate As(III) was determined in vitro using a Methyltransferase Activity Assay Kit (Abcam, Cambridge, UK). Briefly, ArsR^M^, SAM, and arsenious acid were combined; the mixture was adjusted to 50 µL with MT Assay Buffer. Sample Reaction Mix (50 µL) was prepared and added. The absorbance was measured at 570 nm every 30 s for at least 45 min at 37 °C. One unit of methyltransferase activity was defined as the amount of enzyme that generated 1.0 µmol of *S*-adenosyl homocysteine per min at 37 °C.

## Results and discussion

### Isolation and identification of the arsenic-resistant strain M20

**Fig. 1:**
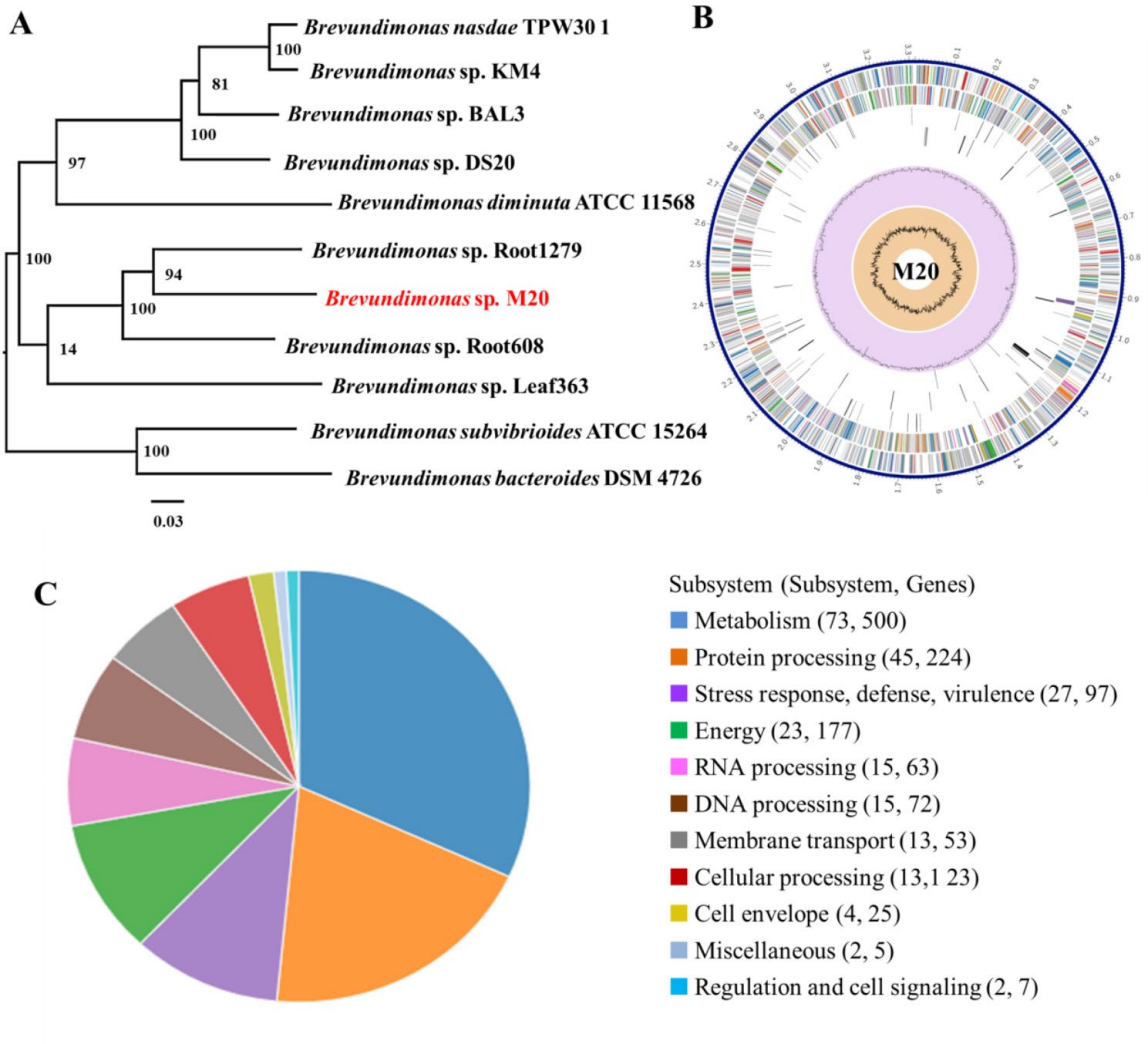
**Identification and genome characteristics of** ***Brevundimonas*** **sp. strain M20.** (A) Phylogenetic tree including strain M20 based on full genome sequences. GenBank accession no. CP041243. (B) PATRIC annotation of strain M20 genome. (C) Subsystem analysis of strain M20 genome

Strain M20 was isolated from aquaculture sewage under the pressure of 30 µM roxarsone, an organic compound of arsenic. MIC analysis revealed that strain M20 was also resistant to inorganic arsenic compounds, such as NaH_2_AsO_3_ and NaH_2_AsO_4_ (Table S3). On the basis of phylogenetic affiliation analysis, strain M20 was identified as *Brevundimonas* sp. (Fig. 1A). *Brevundimonas* sp. is opportunistic pathogens [30]. *Brevundimonas* sp. M20 harbors a 3.315-Mb chromosome (67.56% G + C mol%) (Fig. 1B). The genome annotation included 1,208 hypothetical proteins and 2,052 proteins with functional assignments. The proteins with functional assignments included 758 proteins with Enzyme Commission (EC) numbers, 648 with Gene Ontology (GO) assignments, and 567 proteins that were mapped to Kyoto Encyclopedia of Genes and Genomes pathways (Table S4). Subsystem analysis of PATRIC annotation indicted that strain M20 contains 97 stress response, defense, and virulence genes, and 53 membrane transport-related genes (Fig. 1C).

### Identification of ***ars*** cluster and ***met*** operon

**Fig. 2:**
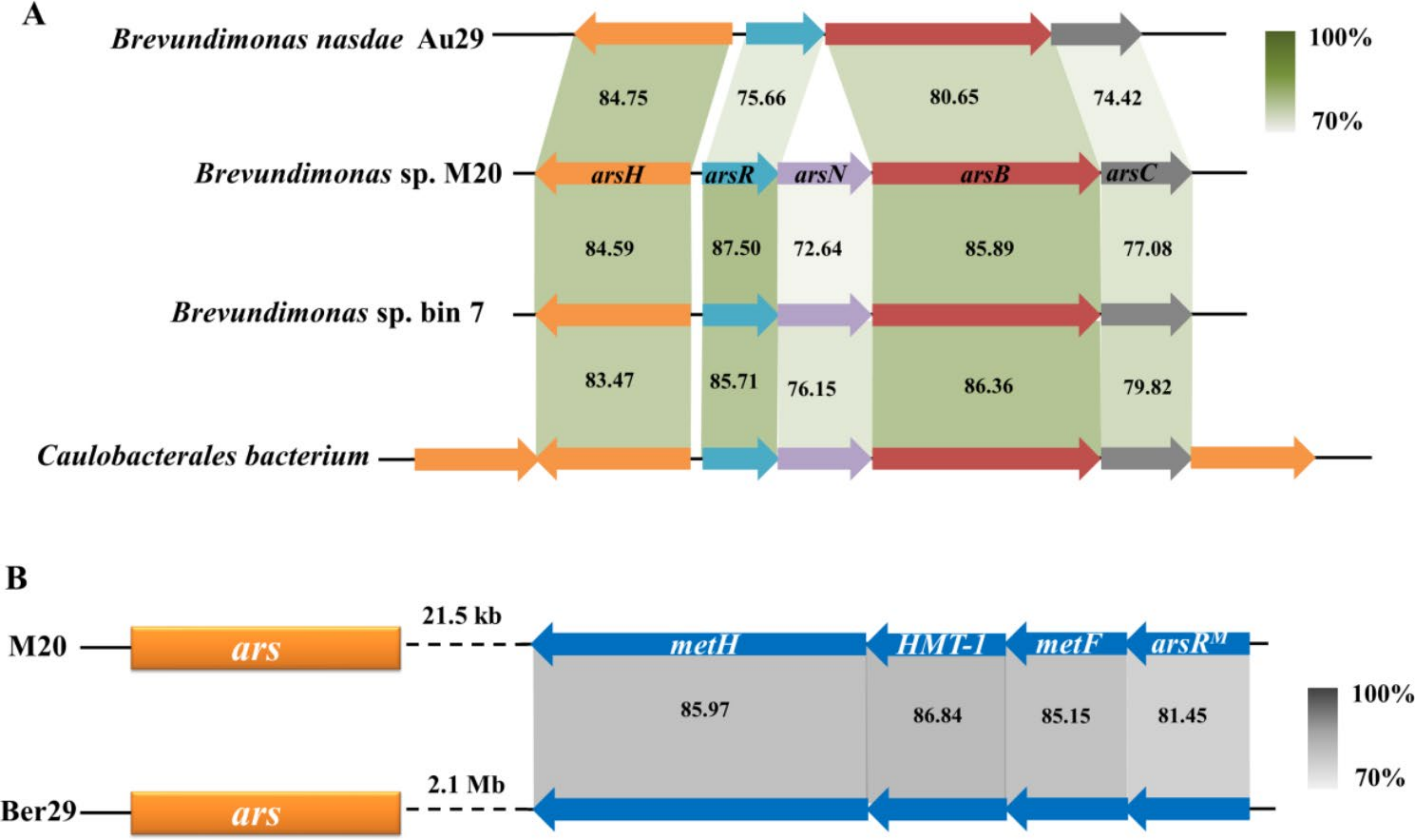
***ars*** **cluster and** ***met*** **operon in** ***Brevundimonas*** **sp. M20.** Physical maps were generated using Easyfig. (A) Linear comparison of *ars* clusters in *Brevundimonas* sp. M20, *Caulobacterales* spp., *Brevundimonas* sp. bin7, and *B. nasdae* Au-Bre29. (B) Linear comparison of *met* operons in *Brevundimonas* sp. M20 and *B. nasdae* Au-Bre29. Block arrows indicate gene length and orientation, and numbers in each box represent nucleotide sequence identity (percent). Distance between *ars* gene cluster and *met* operon is indicated

Genome annotation of *Brevundimonas* sp. M20 revealed a novel *ars* cluster with novel gene arrangement, consisting of the putative arsenic resistance genes *arsH*, *arsR*, *arsN*, *arsB*, and *arsC* (Fig. 2). In this 3,011-bp cluster, *arsH* is an organoarsenical oxidase-encoding gene, which was considered to confer resistance to roxarsone and methylarsenite [20]. *arsR*, encoding a regulatory protein, is responsible for regulating *ars* cluster gene expression. *arsB* and *arsC* are arsenic detoxification genes; arsenate is reduced to arsenite by arsenate reductase (ArsC), followed by efflux of arsenite by the arsenite transporter ArsB [7]. ArsN detoxifies arsenate by acetylation of the α-amino group of arsinothricin [10]. Gene mining was then operated by BLAST analysis. The novel *ars* cluster revealed high similarity with the *ars* cluster in *Caulobacterales* spp. (JAFLCT010000083) and *Brevundimonas* sp. Bin7 (JACVCC01000001), which were not reported in previous researches. To our knowledge, the *ars* cluster in *B. nasdae* strain Au-Bre29 (CP080034)is the only similar one reported so far [13], but it does not contain *arsN* (Fig. 2A).

No arsenic methylation gene was found in the *ars* cluster of *Brevundimonas* sp. M20. However, a 5649-bp *met* operon related to methionine biosynthesis is located downstream of the *ars* cluster. The *met* operon is located 21.5 kb downstream of the *ars* cluster and contains four genes, *arsR*^*M*^ (encoding a transcriptional regulator fused with a methyltransferase), *metF* (encoding methylenetetrahydrofolate reductase), *HMT*-1 (encoding 5-methyltetrahydrofolate-homocysteine methyltransferase), and *metH* (encoding methionine synthase) (Fig. 2B). The DNA sequence of the *arsR* fragment in *arsR*^*M*^ showed very low similarity to the *arsR* gene in the *ars* cluster, although the similarity of the amino acid sequence was 21.78%. The *met* operon showed high similarity with that in *B. nasdae* Au-Bre29, which was isolated in Fujian Province, China, in 2022 [13]. However, the gap between the *ars* cluster and the *met* operon in strains M20 and Au-Bre29 was 21.5 kb and 2.1 Mb, respectively. The function of ArsR^M^ has not yet been explained.

### ArsR ^M^ increased the arsenite resistance of recombinant ***E. coli***

**Fig. 3:**
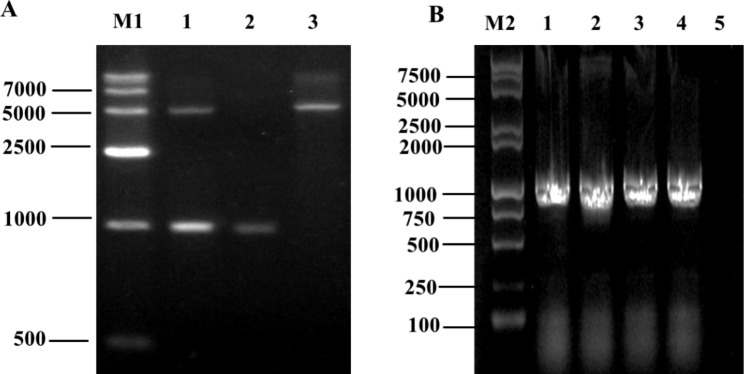
**Construction of** ***Brevundimonas*** **sp. M20 ArsR**^**M**^ **expression strain** ***Escherichia coli*** **ARM3.** (**A**) Expression plasmid construction; lane M1: DL 15,000 DNA Ladder (Solarbio, M1700), 1: pET-arsRM digested by *Nde*I and *Xho*I, 2: *arsR* ^*M*^ fragment amplified by PCR, 3: pET-15b digested by *Nde*I and *Xho*I. Full-length gel is presented in Supplementary Figure S2. (**B**) PCR verification of *E. coli* strain ARM3; lane M2: D 15,000 + 2000 DNA Ladder (TIANGEN, MD116), 1–4: positive transformants, 5: blank control. Full-length gel is presented in Supplementary Figure S3

No arsenic methylation gene was found in the *ars* cluster of *Brevundimonas* sp. M20, while ArsR^M^, encoded by *arsR*^*M*^ of the *met* operon, was identified as a transcriptional regulator fused with a methyltransferase. To analyze the functions of ArsR^M^, the complete *arsR*^*M*^ gene (960 bp) was cloned and inserted into pET-15b (Fig. 3A). The resulting plasmid was transferred into *E. coli* BL21 (DE3), and the resulting strain *E. coli* ARM3 was verified by PCR (Fig. 3B).

After induction by IPTG, the MIC of NaH_2_AsO_3_ for *E. coli* ARM3 was increased to 1.5 mM compared with that in the uninduced control (Table S3). This enhancement indicated that ArsR^M^ likely contributes to arsenite resistance in strain M20. On the basis of similarities with various arsenite methyltransferases in different species (Figure S1), we speculated that the arsenite resistance mediated by ArsR^M^ may be due to arsenite methylation.

### ArsR^M^ is a bifunctional fusion protein

**Fig. 4:**
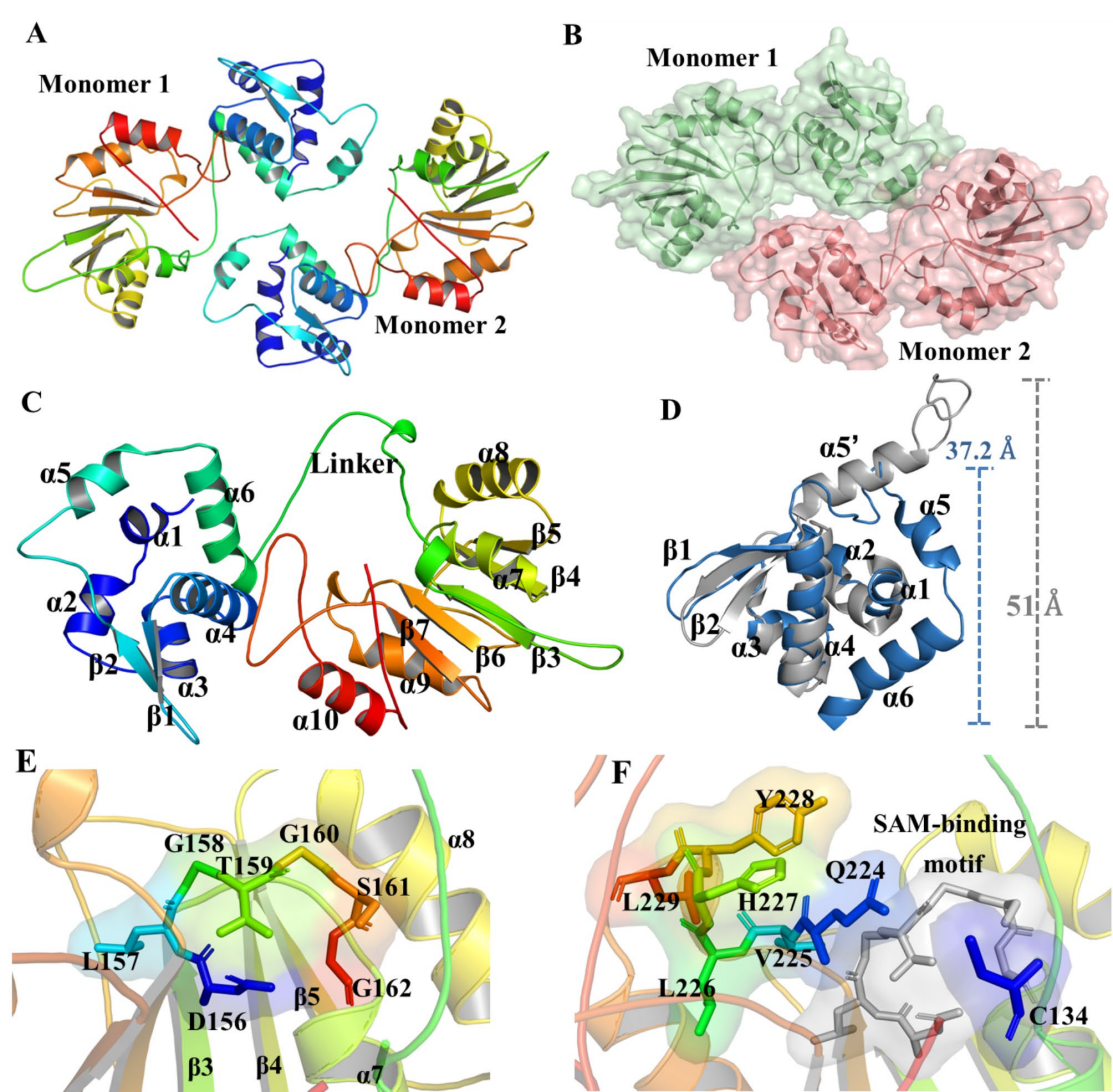
**Structure of ArsR**^**M **^**from *Brevundimonas*sp. M20**. (A) Homologous model of the ArsR^M^ dimer; (B) Different orientations of the two monomers; (C) Structure of ArsR^M^ monomer; (D) Structures of the DNA-binding domain (DBD) of ArsR^M^ and ArsR. ArsR^M^ and ArsR were presented by blue and gray cartoon respectively; (E) *S*-adenosylmethionine-binding motif; (F) As(III)-binding site residues

To further investigate the function of ArsR^M^, its protein structure was homology modeled. The results indicated that ArsR^M^ from *Brevundimonas* sp. M20 forms a dimer (Fig. 4A) and the two monomers adopt different orientations (Fig. 4B). Each monomer contains a transcriptional regulator domain (TRD; α1–α5 and β1–β2), a methyltransferase domain (MTD; α6–α9 and β3–β7), and an 11-amino acid-residue loop linker (residues E111–A121) (Fig. 4C).

Unlike the typical ArsR [Protein Data Bank Code (PDB): 1R1T], which contains five α-helixes and two antiparallel β-sheets [31, 32], the TRD of ArsR^M^ is composed of six short α-helixes and two antiparallel β-sheets. The DNA-binding domain (DBD) in the TRD, which has a shorter diameter of approximately 37.2 Å, compared to that of 51 Å in ArsR (PDB: 3F6O), is composed of six helices (α1–α6) that form the core of the TRD and a *C*-terminal β-hairpin (β1–β2) [33]. TRD of ArsR^M^ contains a longer loop between α5 and α4 compared with 3F6O, and an additional α6 (Fig. 4D). Compared with the tight domain in ArsR, these shortened α-helices in the DBD of ArsR^M^ produce a flexible structure that may lead to a different regulatory mechanism.

The MTD of ArsR^M^ has a mixed structure consisting of α-helices (α6–α9) and β-strands (β3–β7). This domain (residues 122–320) was compared with arsenite methyltransferases in bacteria (including *Thermosediminibacter oceani*, *Streptomyces barringtoniae*, *Chloroflexi* spp., *Alteripontixanthobacter maritimus*, and *Hymenobacter roseosalivarius*); *Homo sapiens* (residues 1–279); and the unicellular red alga *Cyanidioschyzon* sp. (complete protein). A glycine-rich sequence, “DLGTGSG,” is conserved as the hallmark of the SAM-binding motif [31] (Figure S1). This glycine-rich motif has a circular, open shape and is located among α1, α2, β3, β4, and β5 (Fig. 4E). The As(III)-binding site (ABS) has three modular components in ArsR (PDB: 1R1T) [31], while arsenite methyltransferases in different species are variable (Figure S1). The ABS of ArsR^M^, adjacent to the SAM-binding motif, is composed of residue C134 (equivalent to C72 in PDB: 1R1T) and residues in the region of Q224-L229 (Fig. 4F).

### ArsR^M^ binds to its own promoter region

**Fig. 5:**
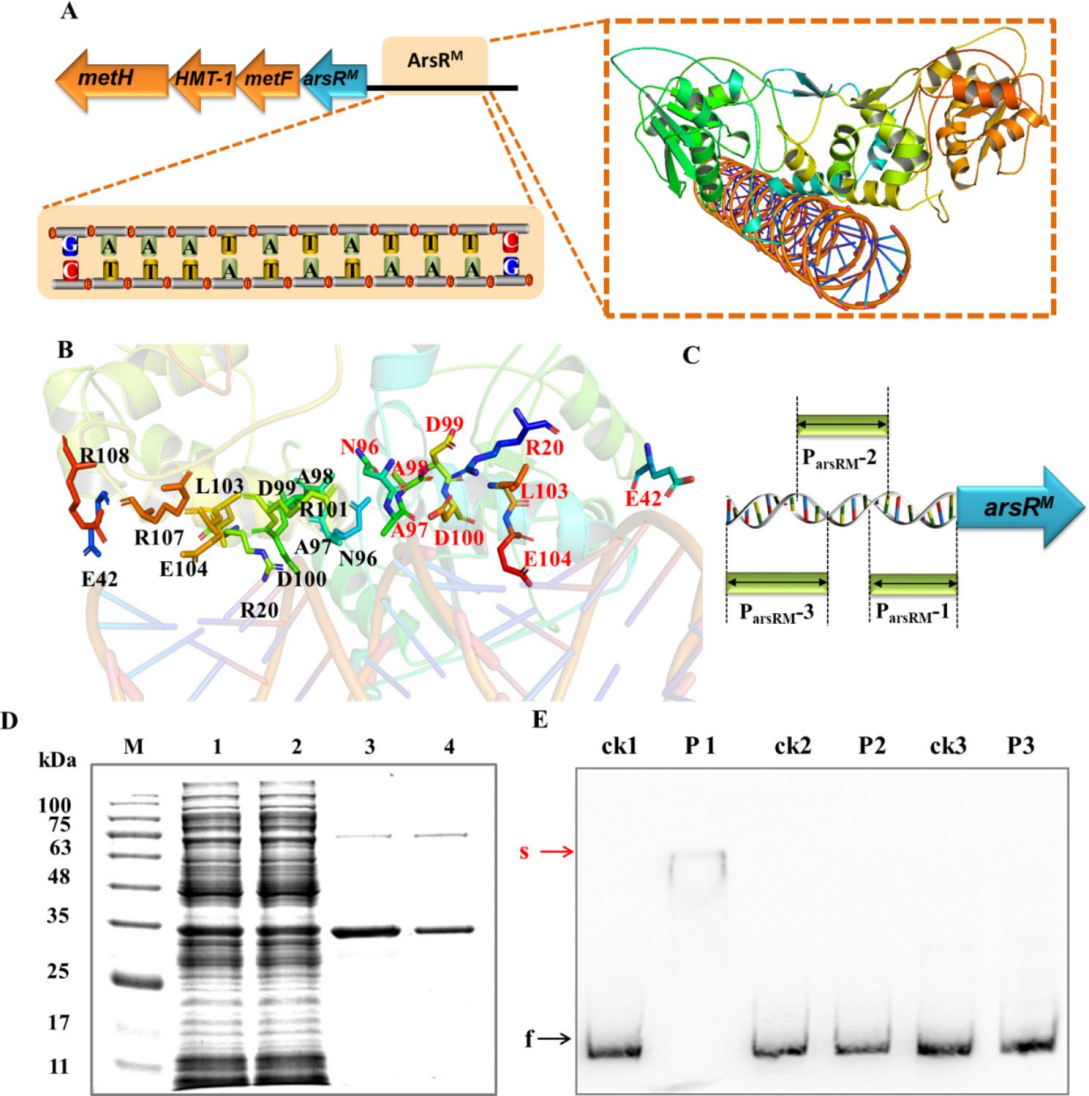
**Verification of the regulatory function of** ***Brevundimonas*** **sp. ArsR** ^**M**^. (A) Interactions between the ArsR^M^ DBD and its potential DNA target. (B) Residues that interact with DNA; red and black labels represent residues of monomer 1 and monomer 2, respectively. (C) Oligonucleotide probes from upstream of *arsR*^*M*^. (D) ArsR^M^ expression in *E. coli* BL21 (DE3). Lanes 1–2: unpurified total protein; 3–4: eluent from Ni-NTA column. Full-length gel is presented in Supplementary Figure S4. (E) Electromobility shift assays of ArsR^M^ and P_arsRM_. Lanes ck1–ck3: probes P_arsRM_-1, P_arsRM_-2, and P_arsRM_-3, respectively, without ArsR^M^; lanes P1–P3: probes P_arsRM_-1, P_arsRM_-2, P_arsRM_-3, respectively, with ArsR^M^. The positions of the free (f) and shifted (s) probes are indicated by black and red arrows, respectively. Full-length gel is presented in Supplementary Figure S5

Molecular docking analysis showed that the TRD of the fusion protein ArsR^M^ from *Brevundimonas* sp. M20 can bind to its own gene promoter region (P_arsRM_). Similar to the DNA binding mechanism of ArsR [32], the dimer forms wing regions and helix α5 (equivalent to α4 in ArsR, PDB: 1GXP) interact with DNA in helix-turn-helix (HTH)–DNA complex structures (Fig. 5A). The DNA-binding sequence of ArsR^M^ in the P_arsRM_ region includes a region that contains the palindromic sequence “CTTTATATAAAG” located upstream of the initiation codon of *arsR*^*M*^ (Fig. 5A). The interacting residues include R20 and E42, and, in helix α5, N96, A97, A98, D99, D100, L103, and E104 (Fig. 5B).

We tested three biotin-labeled probes of the *arsR*^*M*^ promoter region (215-bp long) in EMSAs: P_arsRM_-1 (containing the predicted target sequence), P_arsRM_-2, and P_arsRM_-3 (Fig. 5C). Sufficient quantities of recombinant full-length ArsR^M^–His_6_ protein were produced in *E. coli* BL21 (DE3). The soluble protein was eluted from a Ni-NTA column in buffer containing 150 mM imidazole (Fig. 5D). Probe P_arsRM_-1, containing the predicted binding sequence of ArsR^M^ to P_arsRM_, was shifted in EMSAs by the addition of purified ArsR^M^ (Fig. 5E). This result confirmed the binding of ArsR^M^ to its own promoter region, which might regulate the transcription of the methionine biosynthesis gene cluster in *Brevundimonas* sp. M20.

### ArsR^M^ contributes to the methylation of as(III)

**Fig. 6:**
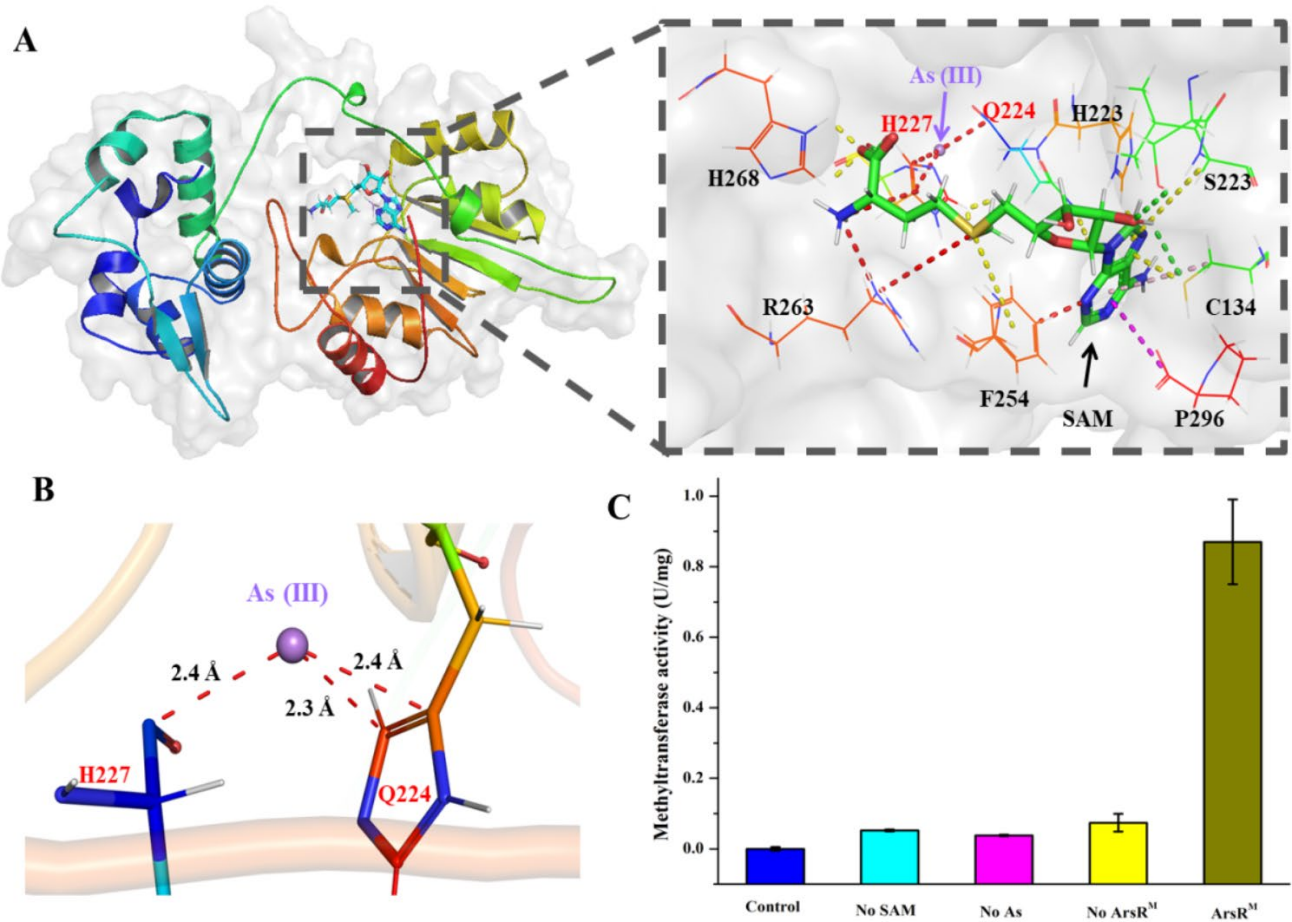
**ArsR**^**M**^**contributes to the methylation of As(III).** (A) Structural overview of ArsR^M^ and interactions between ArsR^M^ and its substrates As(III) and SAM. (B) Interactions of ArsR^M^-bound As(III). Bound arsenic is shown as purple spheres. (C) Methyltransferase activity of ArsR^M^. Control: reaction mixture with ArsR^M^ only; no SAM: reaction mixture without SAM; no AS: reaction mixture without As(III); no ArsR^M^: reaction mixture without ArsR^M^; ArsR^M^: complete reaction mixture including ArsR^M^, SAM, and As(III).

The process of arsenic methylation was originally proposed to be a detoxification mechanism, by which trivalent inorganic arsenic is biotransformed to a less toxic pentavalent methylated form. The MTD of ArsR^M^ was predicted to interact with SAM and trivalent arsenic (Fig. 6A). Amino acid residues C134, H223, Q224, H227, F254, R263, H268, and P296 were identified as the SAM-binding residues (Fig. 6A). Unlike in the arsenite methyltransferases shown in Figure S1 where Cys interacts with As(III), in ArsR^M^, Q224 and Y228 are the As(III) interacting residues, at a distance of 2.3–2.4 Å (Fig. 6B). These distances are similar to those for residues (C174 and C224) in *C. merolae* ArsM (2.2–2.3 Å) [31].

The methyltransferase activity of ArsR^M^ was measured using a Methyltransferase Activity Assay Kit. Activity was detected in the presence of a mixture of SAM, arsenious acid, and ArsR^M^ (Fig. 6C). The results confirmed that arsenious acid can be methylated to monomethylarsonic acid by ArsR^M^ with an activity level of 0.87U/mg.

## Discussion

Arsenic is a metalloid found worldwide [4]. Natural and anthropogenic activities both lead to arsenic accumulation and its transfer into the biosphere [34]. The higher affinity and ability to form metallic compounds make As(III) more toxic than As(V) [2]. In the environment, inorganic arsenic, such as As(V), As(III) [2], are thought to be more toxic than organic arsenic, such as Rox(V)] and Rox(III) [7]. Exposure to arsenic causes diseases, including diabetes, peripheral vascular disease, and cancer [35, 36]. The toxicity, mobility, and fate of arsenic in the environment are influenced by many factors, including its speciation, changes in its speciation, and biogeochemical cycle [2]. In this study, we isolated an arsenic-resistant bacterial strain, *Brevundimonas* sp. M20, which is tolerant to organic and inorganic arsenicals. Genomic annotation indicated the presence of various arsenic-tolerance genes, including those encoding proteins involved in the valence transition of inorganic arsenic and the transformation of organic and inorganic arsenicals, as well as an arsenic efflux pump.

The *ars* cluster is a genetic system to cope with arsenic toxicity in microorganisms. Genes of the *ars* cluster occur in most prokaryotic genomes and are widely distributed in bacterial and archaeal species [37]. *arsRDABC* and *arsRBC* variants are the usual operons found in bacteria [16]. In the present study, we identified a novel *ars* cluster variant in the chromosome of *Brevundimonas* sp. M20; this *ars* cluster contains five genes with the arrangement *arsH–arsR–arsN–arsB–arsC*. Homologous genes of these in *arsHRNBC* cluster are known to participate in organoarsenical oxidation [20, 38, 39], cluster transcriptional repression [40], organoarsenical acetyl transfer [10], formation of arsenite efflux pump[3] and arsenate reduction[16]. Gene functional prediction analyses suggest that this novel *arsHRNBC* cluster contributes to As(III) resistance.

**Fig. 7:**
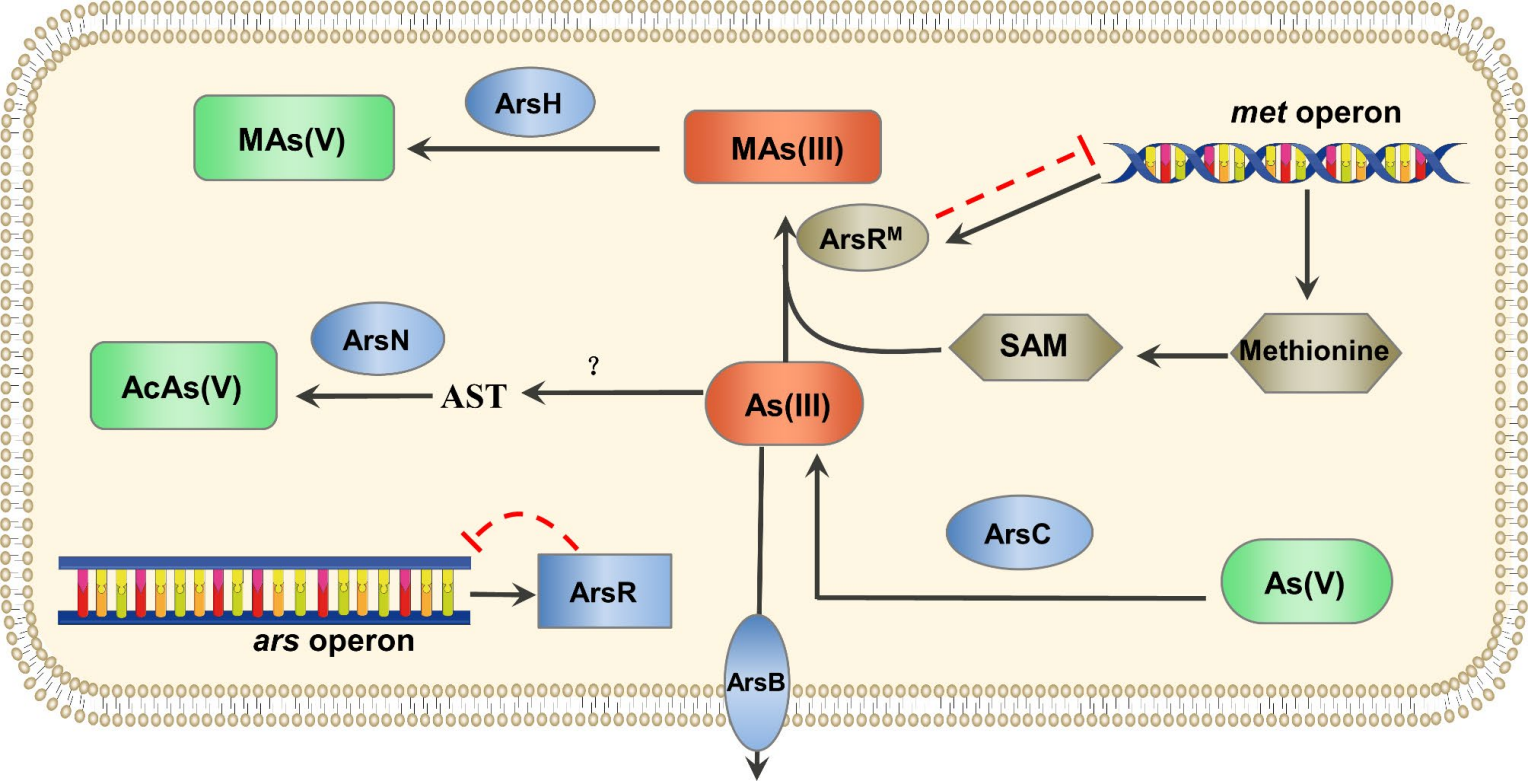
**Mechanisms of arsenic resistance in** ***Brevundimonas*** **sp. M20.** As(III): arsenite; As(V): arsenate; MAs(III): methylarsenite; MAs(V): methylarsenate; AcAs(V): acetylarsenate; AST: arsinothricin; SAM: *S*-adenosylmethionine

The methylation and demethylation of arsenic by microbes are important processes in the arsenic biogeochemical cycle [10], and arsenic methylation results in the natural occurrence of organoarsenic compounds [41]. For methylation, methionine is a universal biological cofactor that transfers its methyl group to substrates such as lipids, proteins, DNA, and other small molecules [10] in the presents of SAM, which is a conjugate of the nucleotide adenosine and amino acid methionine and an essential substrate/cofactor in numerous enzyme-catalyzed reactions, including transmethylation [42]. We found that *Brevundimonas* sp. M20 harbors a methionine biosynthesis gene cluster, the *met* operon, downstream of the *ars* cluster. The first gene, *arsR*^*M*^, encodes a transcriptional regulator fused with a methyltransferase. Molecular simulation and laboratory experiments confirmed the dual function of ArsR^M^. The TRD of ArsR^M^ binds to the palindromic sequence “CTTTATATAAAG” in its own promoter region, P_arsRM_, to regulate expression of the methionine biosynthesis gene cluster, while the MTD of ArsR^M^ has a SAM-dependent arsenic methylation function. ArsR^M^ thus connects methionine biosynthesis and arsenic methylation to provide a precise and efficient pathway for arsenic resistance related to methionine metabolism. Moreover, the organification of As(III), mediated by ArsH along with ArsR^M^, broadens the microbial resistance spectrum of *ars* operons from inorganic to organic arsenic compounds.

The data presented here indicate a contribution to arsenic resistance mediated by the methyltransferase–transcriptional regulator fusion protein ArsR^M^ in *Brevundimonas* sp. M20 (Fig. 7). In this pathway, inorganic arsenic and organic arsenic as As(V) and As(III) are mutually transformed. As(III) can be extruded by the pump ArsB or methylated by ArsR^M^, which also controls the biosynthesis of methionine, a component of SAM. During arsenic methylation, the methyl group of SAM is transferred to As(III) by ArsR^M^ to form methylarsenite [MAs(III)]. MAs(III) is oxidized to MAs(V) by the organoarsenical oxidase ArsH. Elucidation of this novel arsenic-resistance pathway contributes to our understanding of microbial arsenic resistance.

### Electronic supplementary material

Below is the link to the electronic supplementary material.

Additional file 1. Table S1. Bacterial strains and plasmids used in this study. Table S2. Primers used in this study. Table S3. MICs of strains for organic and inorganic arsenics. Table S4. General features of the *Brevundimonas* sp. M20 genome. Figure S1. Multisequence comparison of the MTD domain of ArsR^M^ from *Brevundimonas* sp. M20 with methyltransferase proteins from other species.

## Data Availability

The datasets supporting the conclusions of this article are available from the lead author (Bing Wang:345,807,969@qq.com) upon reasonable request.

## Declarations

### Nucleotide sequence accession number

The complete sequence of the *Brevundimonas* sp. M20 genome has been deposited in GenBank under the accession no. CP041243.
