# Supplementary material for: Bifunctional protein ArsRM contributes to arsenite methylation and resistance in Brevundimonas sp. M20
Source: BMC Microbiol. 2023 May 17;23:134. doi: 10.1186/s12866-023-02876-z (PMC10190100; doi:10.1186/s12866-023-02876-z)
Supplement: Supplementary file 1 — Additional file 1. Table S1. Bacterial strains and plasmids used in this study. Table S2. Primers used in this study. Table S3. MICs of strains for organic and inorganic arsenics. Table S4. General features of the Brevundimonas sp. M20 genome. Figure S1. Multisequence comparison of the MTD domain of ArsRM from Brevundimonas sp. M20 with methyltransferase proteins from other species. [file 12866_2023_2876_MOESM1_ESM.doc]

**Supplementary Materials for**

**Bifunctional protein ArsR^M^ contributes to arsenite methylation and resistance in** ***Brevundimonas* sp. M20**

**Congcong Li^1^, Gongli Zong^2,3^, Wenhui Gao^2,3^, Xi Chen^2,3^, Meixia Tan^2,3^, Jiafang Fu^2,3^, Peipei Zhang^2,3^, Bing Wang^1*†^, Guangxiang Cao^2,3^ ^*†^**

* Correspondence:

345807969@qq.com (Bing Wang);

^1^Shandong Quancheng Test & Technology Limited Company, Ji’nan 250101, China

caoguangxiang@sdfmu.edu.cn (Guangxiao Cao)

^2^Biomedical Sciences College & Shandong Medicinal Biotechnology Centre, Shandong First Medical University & Shandong Academy of Medical Sciences, Ji’nan 250062; ^3^NHC Key Laboratory of Biotechnology Drugs (Shandong Academy of Medical Sciences), Ji’nan 250117 Shandong, China

This PDF file includes:

Supplementary Text

Figures. S1-S5

Tables S1 to S4

**Table S1** Bacterial strains and plasmids used in this study

| **Strains or plasmids** | **Relevant description** | **Reference** | |  |
| --- | --- | --- | --- | --- |
| **Strains** |  | |  | |
| *Brevundimonas* sp. M20 | Arsenic-resistant strain with an *ars* cluster and a *met* operon | | This study | |
| *E. coli* DH5α | Strain used for gene clone | | Sangon | |
| *E. coli* BL21(DE3) | Strain used for protein expression | | Sangon | |
| **Plasmids** |  | |  | |
| pMD18-T | General cloning vector | | Takara | |
| pET-15b | Plasmid for protein expression and purification | | This lab | |

**Table S2** Primers used in this study

| Primer ID | | Primer sequence (5′ →3′) |
| --- | --- | --- |
| **For *arsR^M^* expression and PCR verification** | | |
| arsR^M^-His-F | *catatg*ATGTCGTTGAGCGCTGATCAAACTG (*Nde* I) | |
| arsR^M^-His-R | *ctcgag*Tcatgccgctttcgctttcgtctgaac (*Xho* I) | |
| **For EMSA analysis** | | |
| P_arsRM_-1 | | *ggctttatataaagatatccttatatgatttggcaaggaaaaagcgcggggcggcagcggcgggcgaacctggagcccgtaggggattccgctttcgcccgaccctc |
| P_arsRM_-2 | | *gctttcgcccgaccctccggcgcgacccgttctgtgtttcaatccggaaacaaaaatgcgctggccagacggggcggacgg |
| P_arsRM_-3 | | *tgtgtttcaatccggaaacaaaaatgcgctggccagacggggcggacgggcacactctggcgctgtcgatcacccgtatgcggaagttcttcg |

**Table S3** MICs of strains for organic and inorganic arsenics

| **Strains** | Roxarsone | NaH_2_AsO_3_ | NaH_2_AsO_4_ |
| --- | --- | --- | --- |
| *Brevundimonas* sp. M20 | 1.25 mM | 4.5 mM | 5.5 mM |
| *E. coli* ARM3 (with IPTG) | / | 1.5 mM | / |
| *E. coli* ARM3 (no IPTG) | / | < 0.25 mM | / |
| *E. coli* BL21(pET-15b, with IPTG) | / | < 0.25 mM | / |

/: Not measured

**Table S4.** General features of the *Brevundimonas* sp. M20 genome

| Feature | Genome |
| --- | --- |
| Total number of base pairs | 3,315,348 bp |
| G+C content (%) | 67.56 |
| Total genes | 3,260 |
| tRNA | 48 |
| Repeat Regions | 19 |
| rRNA | 6 |
| Hypothetical proteins | 1,208 |
| Proteins with functional assignments | 2,052 |
| Proteins with EC number assignments | 758 |
| Proteins with GO assignments | 648 |
| Proteins with KEGG Pathway assignments | 567 |
| Proteins with PATRIC genus-specific family (PLfam) assignments | 2,369 |
| Proteins with PATRIC cross-genus family (PGfam) assignments | 2,495 |
| Antibiotic Resistance | 27 |
| Transporter (TCDB) | 3 |
| Virulence Factor (VFDB) | 1 |
| Virulence Factor (Victors) | 2 |

Abbreviations: EC, Enzyme Commission; GO, Gene Ontology database; KEGG, Kyoto Encyclopedia of Genes and Genomes.


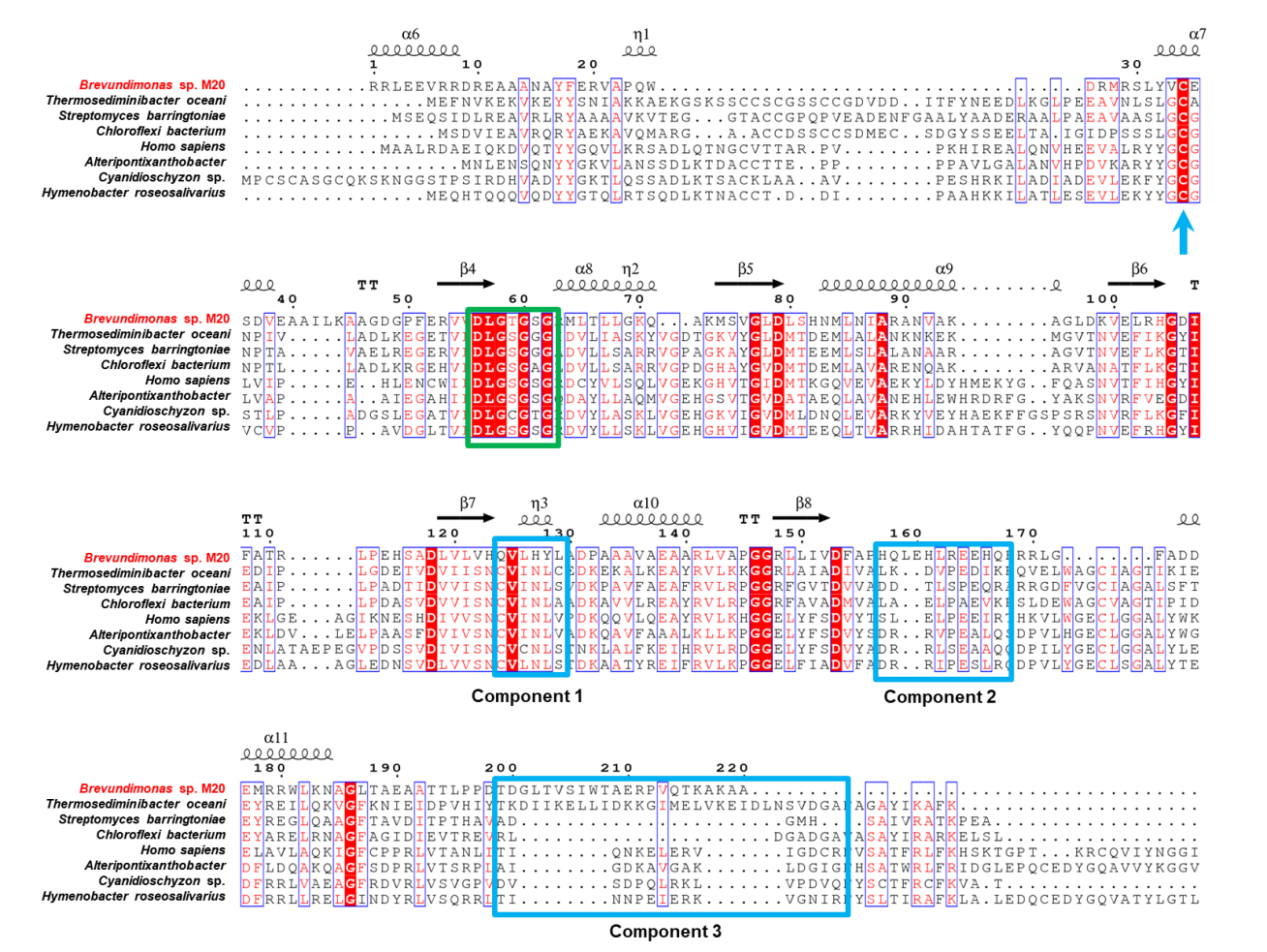


**Figure S1.** Multisequence comparison of the MTD domain of ArsR^M^ from *Brevundimonas* sp. M20 with methyltransferase proteins from other species. *Brevundimonas* sp. M20: WP_019848462.1 (residues 101–220); *Thermosediminibacter oceani*: ADL07380.1 (272 residues, complete protein); *Streptomyces barringtoniae*: WP_228624381.1 (250 residues, complete protein); *Chloroflexi* spp.: TMG71448.1 (248 residues, complete protein); *Homo sapiens*: NP_065733.2 (residues 1–279); *Alteripontixanthobacter maritimus*: RDC60484.1 (residues 1–274); *Cyanidioschyzon* sp.: ACN39191.1 (282 residues, complete protein); *Hymenobacter roseosalivarius*: SMB81317.1 (residues 1–275). The green box indicts the SAM-binding motif; blue boxes and arrows indicate the site and essential residues for As(III)-binding.


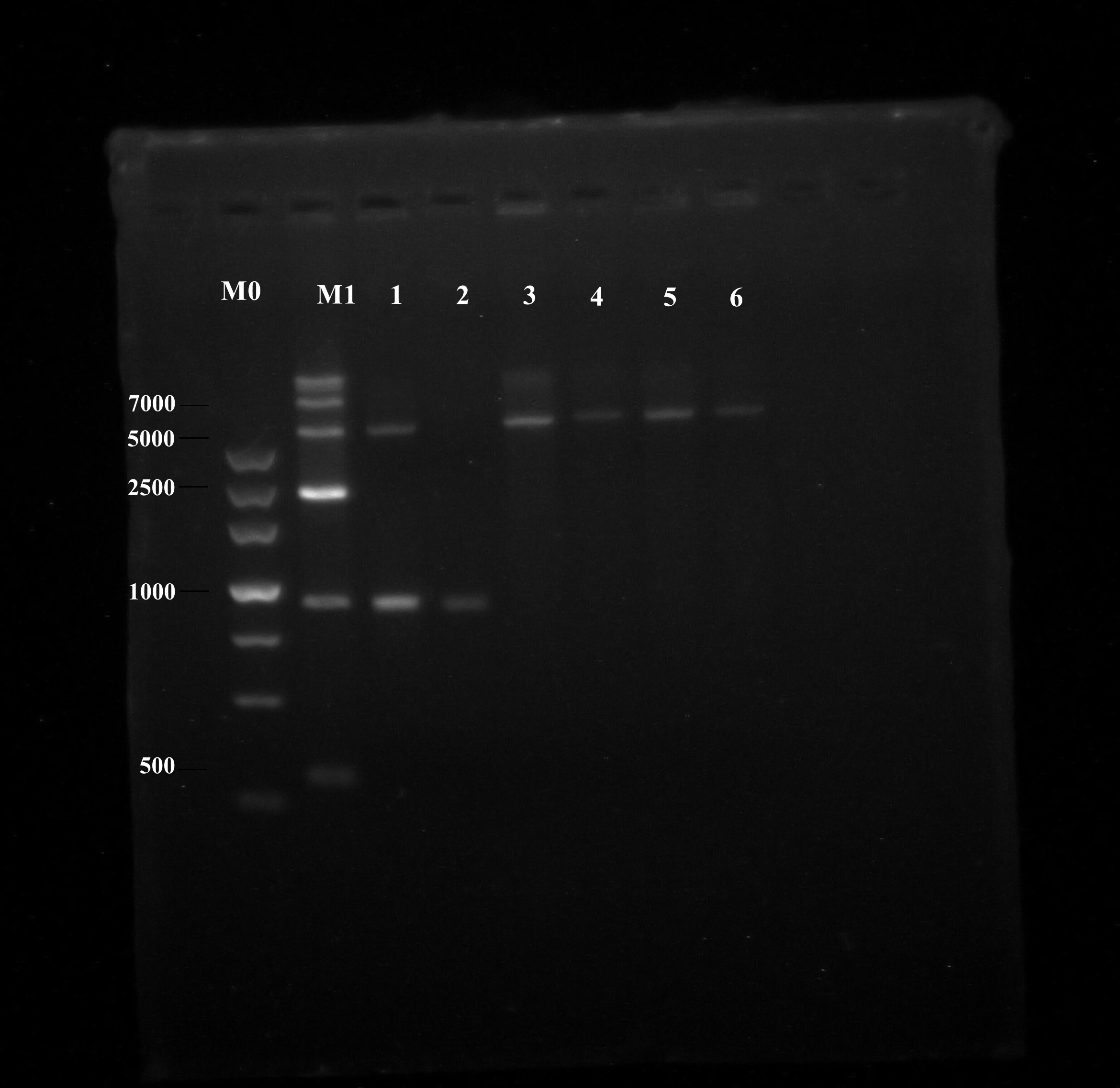


**Figure S2.** Full-length gel of Figure 3A. Figure 3A in manuscript was cropped from M1 to lane 3. Repeat pET-15b controls (lane 4-6) and lane with redundant DNA Ladder III were cropped out.


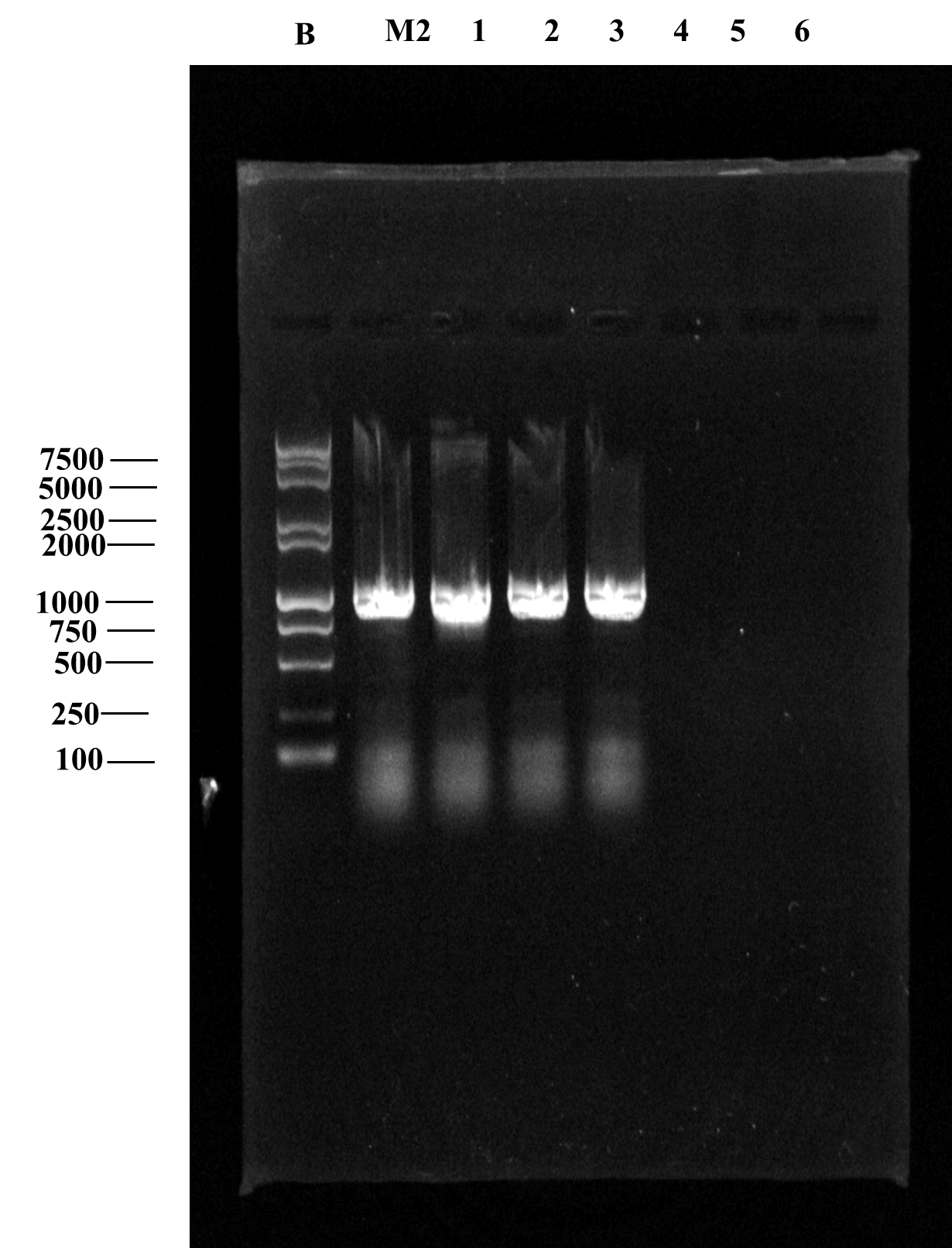


**Figure S3.** Full-length gel of Figure 3B. Figure 3B in manuscript was cropped from lane1 to lane 5, lane 6 was cropped out.


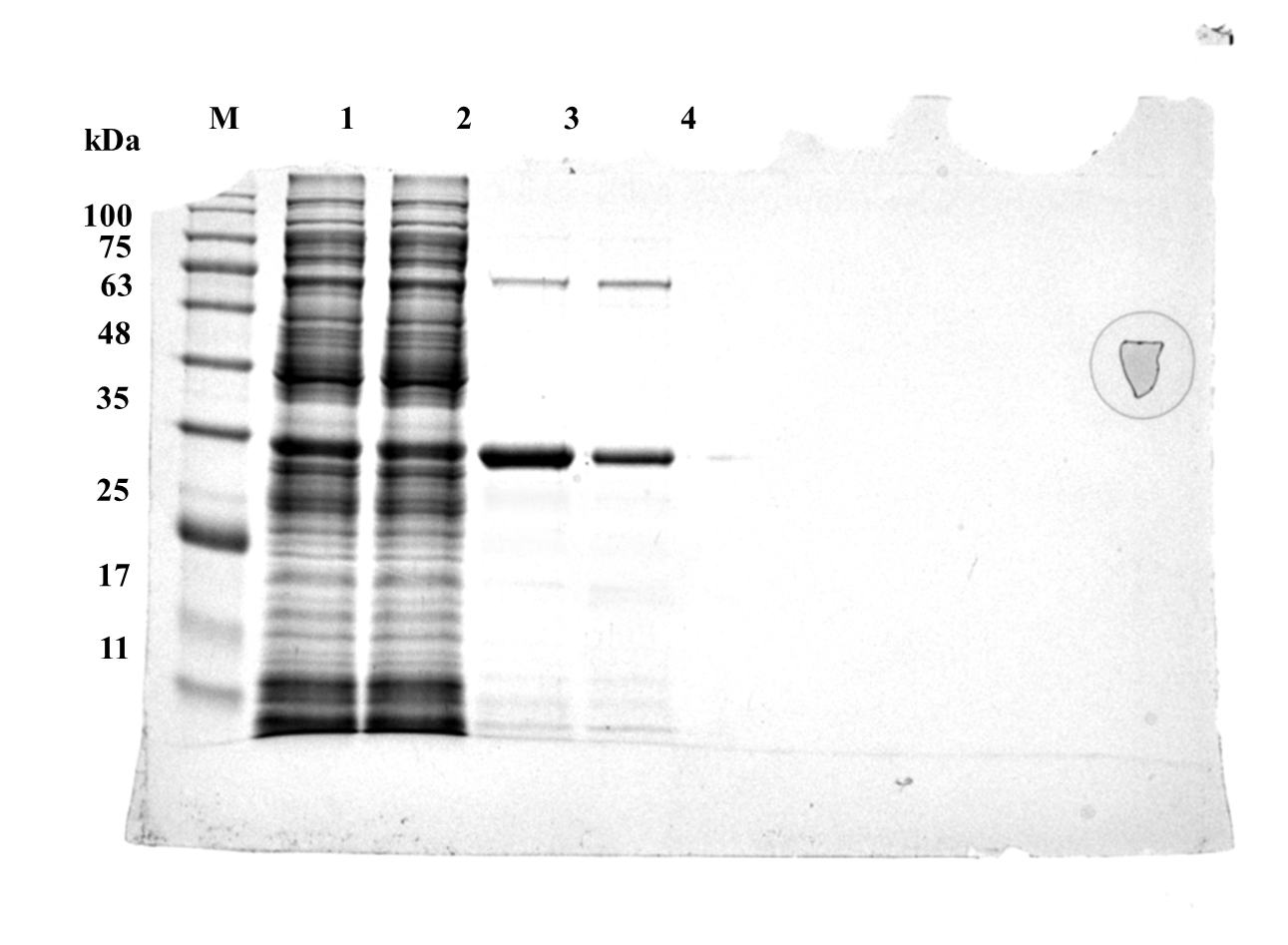


**Figure S4.** Full-length gel of Figure 5D. Figure 5D in manuscript was cropped from lane M to lane 4, blank lanes were cropped out.


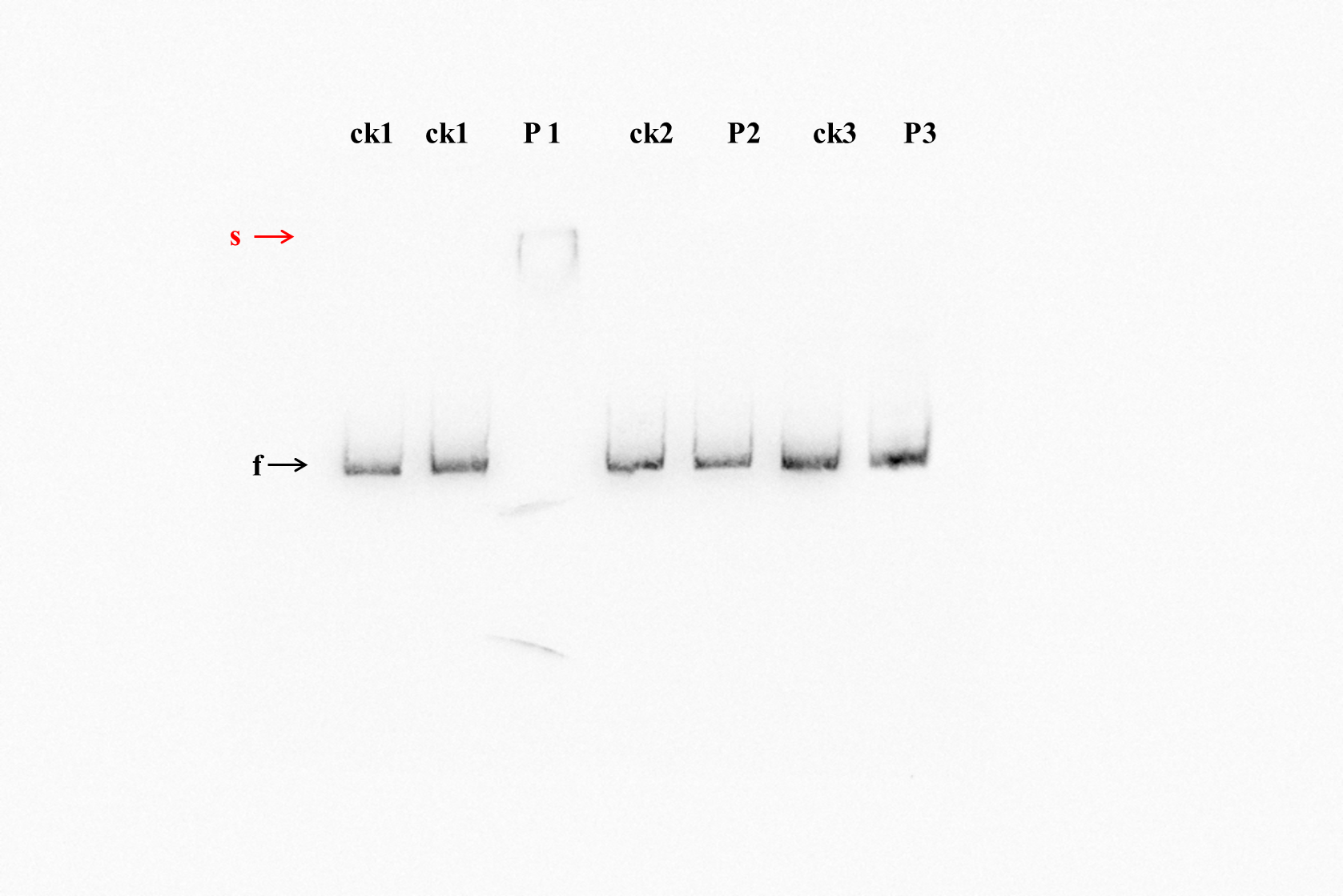


**Figure S5.** Full-length gel of Figure 5E. Figure 5E in manuscript was cropped from lane ck1 to lane p3, repeated lane ck1was cropped out.
